# Membrane Transporter of Serotonin and Hypercholesterolemia in Children

**DOI:** 10.3390/ijms25020767

**Published:** 2024-01-07

**Authors:** Dinara Sadykova, Razina Nigmatullina, Karina Salakhova, Evgeniia Slastnikova, Liliya Galimova, Chulpan Khaliullina, Ildaria Valeeva

**Affiliations:** 1 Department of Hospital Pediatrics, Kazan State Medical University, 420012 Kazan, Russia; karina.salakh@mail.ru (K.S.); e.slastnikova@mail.ru (E.S.); lilu1@inbox.ru (L.G.); chulpandanilevna@yandex.ru (C.K.); 2 Department of Normal Physiology, Kazan State Medical University, 420012 Kazan, Russia; razinar@mail.ru; 3 Children’s Republican Clinical Hospital, 420138 Kazan, Russia; 4 Central Research Laboratory, Kazan State Medical University, 420012 Kazan, Russia; valeeva.ildaria@yandex.ru

**Keywords:** serotonin, SERT, cardiovascular diseases, familial hypercholesterolemia, children, vascular remodeling

## Abstract

The serotonin membrane transporter is one of the main mechanisms of plasma serotonin concentration regulation. Serotonin plays an important role in the pathogenesis of various cardiovascular diseases, stimulating the proliferation of smooth muscle cells, key cells in the process of hypertrophic vascular remodeling. Vascular remodeling is one of the leading prognostically unfavorable factors of atherosclerosis, the main manifestation of familial hypercholesterolemia. Familial hypercholesterolemia is one of the most common genetically determined lipid metabolism disorders and occurs in 1 in 313 people. The aim of our study was to investigate the levels of plasma and platelet serotonin, 5-hydroxyindoleacetic acid, and membrane transporter in a cross-sectional study of two pediatric groups, including patients with familial hypercholesterolemia and the control group, which consisted of apparently healthy children without cardiovascular diseases. The study involved 116 children aged 5 to 17 years old. The proportion of boys was 50% (58/116) and the average age of the children was 10.5 years (CI 2.8–18.1). The concentrations of serotonin in blood plasma and platelets and 5-hydroxyindoleacetic acid were higher in children with familial hypercholesterolemia than in the controls. The concentration of the serotonin transporter in platelets in healthy children, compared with the main group, was 1.3 times higher. A positive correlation was revealed between the level of serotonin (5-HT and PWV: *ρ* = 0.6, *p* < 0.001), its transporter (SERT and PWV: *ρ* = 0.5, *p* < 0.001), and the main indicators of arterial vascular stiffness. Our study revealed the relationship between high serotonin and SERT concentrations and markers of arterial stiffness. The results we obtained suggest the involvement of serotonin and SERT in the process of vascular remodeling in familial hypercholesterolemia in children.

## 1. Introduction

Serotonin membrane transporter (SERT) belongs to the family of Na^+^/Cl^−^-dependent solute transporters (SLC6) [1]. SERT provides a saturable serotonin (5-HT) reuptake mechanism from the synaptic cleft [2]. The structure and role of SERT in the presynaptic membrane of serotonergic synapses of the CNS, where it performs the function of reuptake and transfer of serotonin to the presynaptic terminal, have been well studied. Being a sodium-dependent membrane protein, SERT modifies the effect of serotonin on the receptors of the postsynaptic membrane. The concentration of serotonin in the synaptic contact depends on the activity and density of the carrier [1,3].

Being part of the platelet membrane [4], SERT ensures the transport of serotonin from blood plasma into platelets and its storage in dense granules using a vesicular monoamine transporter [2] or cleavage by intracellular monoamine oxidase (MAO) [1]. It has been shown that increased levels of serotonin in blood plasma correlate with a decrease in the density of transporter molecules on the plasma membrane of platelets [5]. Thus, a significant increase in SERT in platelets was detected in children from 1 month to 2 years old with congenital heart defects complicated by pulmonary arterial hypertension, and it was accompanied by a decrease in the concentration of serotonin. The authors of the study associated this phenomenon with the failure of the SERT function [6].

SERT, participating in the regulation of systemic serotonin homeostasis, is part of various tissues of the human body. It was found in the plasma membranes of smooth muscle cells [7], cardiomyocytes [8], pulmonary endothelium [9], and placental epithelium [10]. The molecular structure of SERT is the same in all of the cell types [11]. However, the functional role of SERT in the membranes of muscle and endothelial cells has not been studied enough. It has been revealed that SERT in platelets ensures the deposition of serotonin from blood plasma. This mechanism is associated with a change in the concentration of serotonin in the blood, while up to 95% of serotonin is stored in platelets and less than 5% circulates freely in plasma [12,13].

Serotonin homeostasis in the human body is also ensured by conformational changes in SERT. Thus, when the concentration of serotonin in the blood plasma is up to 0.1 µM, SERT is in a “sleeping” state, which corresponds to phase 0. An increase in the level of free serotonin activates SERT and stimulates a change in its conformation from an inactive, inward-facing conformation to a new outward position, which is accompanied by a higher affinity for serotonin that significantly activates its transport. These SERT changes are described as phase 1. Abnormally high levels of 5-HT in blood plasma lead to a transition to phase 2, which is characterized by disruption of the membrane transporter and reversion to an inward-facing conformation with low affinity for serotonin [14].

Recent studies have shown that conformational changes in SERT depend on more than the concentration of serotonin in the blood plasma [14]. Cholesterol is also able to interact with SERT, changing its configuration and function [15]. This lipid induces a transient outward-facing conformation, increasing the membrane transporter’s affinity for serotonin [16]. Scanlon et al. studied the disruption of the functional activity of SERT in human embryonic kidney cells by changing the cholesterol content in them. Depletion of the level of cholesterol led to a decrease in the activity of the membrane transporter through a change in its conformation, which reduced the affinity of SERT for serotonin and, accordingly, the rate of its transport [17].

Serotonin is one of the main neurotransmitters that performs a wide range of functions in the body due to the wide variety of receptors from 5-HT1 to 5-HT7, which are both ionotropic (5-HT3) and metabotropic (5-HT2, 5-HT4) [18,19]. The presence of a large number of serotonin receptors in the cardiovascular system [20,21,22] determines the important role of serotonin in the pathogenesis of various cardiovascular diseases (CVDs) [23]. By acting on the walls of blood vessels, 5-HT promotes thrombosis, mitogenesis, proliferation of vascular smooth muscle cells (VSMCs), and the formation of macrophage foam cells [24,25]. The 5-HT2A receptor belongs to a large family of transmembrane receptors (GPCRs). Molecular dynamics simulations showed that cholesterol is involved in GPCR activation through two mechanisms: direct (changes in the structure of the receptor due to direct interaction with cholesterol) and indirect (changes in the properties of the 5-HT2A receptor membrane due to changes in its physical properties that modulate the structure and dynamics of the receptor) [26].

The involvement of serotonin and SERT in the initiation of cardiovascular diseases is confirmed by recent studies, which were conducted both in vitro and in experimental animal models [2,24]. The experimental data presented are supported by only a small number of clinical studies [27,28]. An ideal model for studying the influence of serotonin and membrane transporters on the atherosclerotic process of vascular changes would be familial hypercholesterolemia.

Familial hypercholesterolemia is one of the most common genetically determined lipid metabolism disorders [29,30], accompanied by increased concentrations of low-density lipoproteins (LDL-cholesterol) in blood plasma, early development of atherosclerotic lesions of the vessels, and a high risk of premature cardiovascular diseases [31]. According to the results of a meta-analysis, which included data from 11 million people, the prevalence of the heterozygous form of familial hypercholesterolemia is 1 case per 313 people [32]. There are approximately 35 million people living in the world today with familial hypercholesterolemia, and 6.8–8.5 million of them are children; one child is born with a heterozygous form of familial hypercholesterolemia every minute worldwide [33,34].

The heterozygous form of familial hypercholesterolemia is asymptomatic during the first decades of life, and therefore there are no clinical manifestations of the disease in contrast to the homozygous form, which is characterized by the presence of specific symptoms at an early age (xanthomas and xanthelasmas, lipoid arch of the cornea) [33]. However, the results of two large studies conducted in the USA demonstrated that the atherosclerotic process already begins in childhood. It was found that the degree of coverage of the surface of the intima with atherosclerotic lesions was associated with increased concentrations of total cholesterol (TC), LDL-cholesterol, and triglycerides (TGs), as well as a lower concentration of high-density lipoprotein (HDL-cholesterol) [35].

A number of studies have shown that hypercholesterolemia can cause loss of elasticity and increased stiffness of arterial vessels, leading to an increase in pulse wave velocity (PWV) due to rapid propagation in stiff arteries [36,37]. Arterial stiffness is closely associated with structural changes in the arteries, characterized by thickening of the intima-media complex, which is also a preclinical marker of early vascular damage [38]. Changes in the elastic properties of the arteries may indicate a functional disorder long before the onset of clinical lesions. The study of arterial stiffness is quite widespread among adult patients [39,40], while in pediatrics, this diagnostic method, due to objective circumstances, is used much less frequently. The study of changes in arterial wall stiffness is performed as part of ambulatory blood pressure monitoring (ABPM) using special systems. This procedure is quite time-consuming, cannot be used in children with small shoulder girths, and is usually available only in large medical centers. In this regard, the use of ABPM with arterial stiffness measurement in pediatric practice is limited.

Despite the fact that 5-HT has a wide range of possible effects on the cardiovascular system, the currently available literature data do not provide a complete explanation of the pathogenetic mechanisms of the influence of serotonin on the development of cardiovascular pathology, including atherosclerotic vascular lesions. Further studies of the clinical and physiological effects of 5-HT, the membrane transporter of serotonin, and their role in the pathogenesis of CVD in children are necessary.

The aim of the study is to evaluate changes in the level of the membrane transporter of serotonin, serotonin, and its metabolite 5-hydroxyindoleacetic acid (5-HIAA) in blood plasma and platelets as an early (preclinical) marker of vascular remodeling. The results of this kind of research are practically not found in the specialized scientific literature. The data obtained will contribute to a better understanding of the mechanism of vascular remodeling in hypercholesterolemia at both the molecular and pathophysiological levels.

## 2. Results

### 2.1. Laboratory and Instrumental Characteristics of the Study Groups

Routine laboratory examination methods (complete blood count, biochemical blood test, and coagulogram) did not reveal statistically significant differences between the compared groups. But the lipid profile indicators, as expected, differed. The concentration of LDL-cholesterol in children with familial hypercholesterolemia was 2 times higher than in children in control, *p* < 0.001. The average level of HDL-cholesterol in the main group was lower compared to healthy children, *p* < 0.05. Lipidogram results are presented in Table 1.

The presence of familial hypercholesterolemia was accompanied by a significant increase in PWV—minimum, mean, and maximum values. We did not find statistically significant differences in the minimum augmentation index (AIxmin) (*p* = 0.057) and the maximum augmentation index (AIxmax) (*p* = 0.179). At the same time, statistically significant differences were identified for the mean augmentation index (AIxmean) in patients with familial hypercholesterolemia compared with their healthy peers (Me—39.5 [Q1–Q3 −47–−35] and Me—51 [Q1–Q3 −58–−45], *p* < 0.001). In children from the main group, the ambulatory stiffness index (AASI) was 25% higher. The results of ABPM are presented in Table 2.

**Figure 1:**
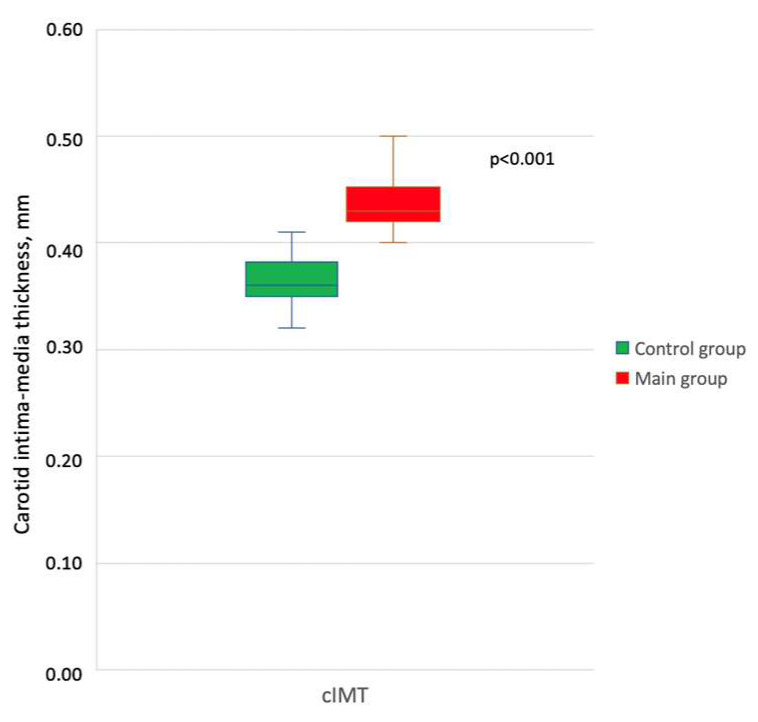
Comparison of the thickness of the intima-media complex of the common carotid artery in the study groups.

All children underwent measurement of carotid intima-media thickness (cIMT), which is used for early diagnosis of atherosclerosis in adult patients with familial hypercholesterolemia, while in pediatrics, despite being non-invasive and highly informative, it is used much more rarely. An increase in cIMT was found in children with familial hypercholesterolemia (Me—0.45 [Q1–Q3 0.42–0.48] mm) compared with the control group (Me—0.4 [Q1–Q3 0.37–0.4] mm), *p* < 0.001 (Figure 1).

### 2.2. Main Results and Their Comparison

Plasma 5-HT concentration (expressed in pmol/mL) in patients with familial hypercholesterolemia was 1.5 times higher than in controls (Me—96.7 [Q1–Q3 24.2–175.2] vs. Me—66.2 [Q1–Q3 42.4–79.2] pmol/mL, *p* < 0.001).

The concentration of serotonin in platelets, both in total quantity and in terms of one platelet, was higher in patients in the main group than in the control group, *p* < 0.05.

The mean concentrations of 5-HIAA, the main metabolite of serotonin, in the main group were 1.3 times higher than in the control group (27.2 pmol/mL vs. 20.9 pmol/mL, respectively, *p* < 0.001).

Statistically significant differences were obtained comparing the average values of the membrane serotonin transporter. The mean SERT concentration in children with familial hypercholesterolemia was 0.04 ng/mL, while in the control group it was 0.03 ng/mL, which corresponds to a 25% decrease in its concentration, *p* < 0.001. The data are presented in Table 3.

### 2.3. Spearman Correlation

Our study revealed a strong positive correlation between the concentration of serotonin in blood plasma and its concentration in platelets, with 5-HIAA and SERT, and a positive correlation of moderate strength with such instrumental markers of arterial stiffness as cIMT and PWVmin, PWV, PWVmax, and AASI (Table 4).

A positive correlation was also found between the concentration of serotonin in the blood plasma and the main indicators of the lipid profile: TC and LDL-cholesterol, and a negative correlation with the level of HDL-cholesterol (Table 5).

In addition, a strong and moderate relationship was found between the concentration of serotonin in platelets and the number of platelets, the level of 5-HIAA, PWVmin, PWV, PWVmax, AASI, and cIMT (Table 4). We determined the presence of a connection between the level of 5-HIAA, the main markers of arterial stiffness, and lipid profile parameters in the process of correlation analysis (Table 4 and Table 5). The study also determined a positive correlation between the concentration of the serotonin transporter in platelets and PWVmin, PWV, PWVmax, and AASI. A strong direct relationship was found between membrane serotonin transporter levels and protein (*ρ* = 0.6, *p* < 0.001).

## 3. Discussion

Our study showed a connection between the level of serotonin, the membrane transporter of serotonin, and atherosclerotic changes in the vascular wall, which we identified by measuring the stiffness of arterial vessels and the thickness of the intima-media complex of the common carotid artery. We hypothesize that serotonin itself and its membrane transporter may be involved in the pathogenesis of vascular remodeling and the formation of atherosclerosis in children.

The serotonin membrane transporter is a protein that is the main mechanism for regulating the concentration of serotonin in blood plasma [1,12]. SERT has been found in the plasma membranes of VSMCs, where it is involved in systemic serotonin homeostasis [7]. With an increase in the concentration of 5-HT in blood plasma, the levels of SERT in the platelet plasma membrane increase, and thereby the uptake of serotonin increases [1]. The present study shows that SERT levels in children with familial hypercholesterolemia were 1.3 times higher than in children without CVD. This is consistent with the data of Kim et al., which demonstrated an increase in platelet aggregation and activation in adult patients with coronary heart disease as serotonin concentration increased, due to an increase in the level of SERT on the plasma membrane [41]. In another study, Ziu et al. determined platelet function after a 24-h increase in serotonin levels in mice in vitro and in vivo. They found an initial increase in SERT-mediated 5-HT uptake with increased platelet aggregation, followed by a loss of SERT at the platelet plasma membrane and a decrease in 5-HT uptake [2]. The researchers hypothesized that abnormally high plasma levels of serotonin lead to SERT dysfunction. Despite this, even at the highest plasma levels of 5-HT, there are always a small number of SERT molecules on the platelet plasma membrane that still continue to remove 5-HT from the plasma, but at a slower rate. They are active, probably, until the physiological level of plasma serotonin is reached [1,5]. In the present study, we did not observe a loss of function of the serotonin membrane transporter. We assume that such data were obtained due to the fact that the concentration of serotonin in the blood plasma in children did not reach abnormally high values due to the “short duration” of the disease. It is possible that SERT activity is supported by elevated cholesterol levels in the blood plasma, which is consistent with data obtained in vitro and in experimental animal models [16,17]. This correlation has been studied partially in children with congenital heart defects complicated by pulmonary arterial hypertension. Mindubaeva et al., examining such patients aged 1 month to 2 years, revealed a significant increase in SERT in platelets with a simultaneous decrease in serotonin levels in them. The authors indicated that the determination of SERT concentration in platelets may serve as a biomarker for the presence of pulmonary arterial hypertension in children with congenital heart defects [6].

Back in 1999, Vikenes et al., based on the results of their work, reported that high levels of serotonin in platelet-rich plasma are associated with coronary heart disease and the progression of cardiovascular diseases [27]. As with SERT in our study, patients had higher 5-HT levels than controls (ratio: 1.5:1). Also, in our work, children with familial hypercholesterolemia had higher levels of 5-HT in platelets compared to healthy children, both in total and per platelet. We assume that a possible reason for the increase in serotonin concentration in platelets in patients with familial hypercholesterolemia compared to healthy children may be its increased synthesis in the microserotonergic systems of the body [42]. It has been proven that in peripheral arteries there is a local 5-hydroxytryptaminergic system capable of synthesizing serotonin [43]. Studies on the relationship between serotonin and atherosclerosis are extremely scarce. Ma et al. demonstrated in animal models that sarpogrelat, as a 5-HT2A receptor antagonist, can inhibit the development of atherosclerosis [24]. Sarpogrelat reduced the volume of plaques in the coronary arteries in the group of patients with type II diabetes mellitus [44].

5-HT, being a mitogen of VSMCs, promotes their growth in arterial walls [45,46]. This may subsequently lead to vascular remodeling [47]. A manifestation of vascular remodeling is an increase in cIMT. The results of our study confirmed this statement: cIMT in children with familial hypercholesterolemia was increased compared to the control group, which correlated with plasma serotonin levels. It has been suggested that VSMC-mediated remodeling of structural extracellular matrix components such as collagen and elastin may lead to increased arterial stiffness [48], an indicator that is a traditional independent predictor of cardiovascular risk in patients with familial hypercholesterolemia [49]. Serotonin, through 5-HT2A and 5-HT2B receptors, initiates the activation of fibroblasts, which leads to the proliferation of connective tissue and increased vascular stiffness [50]. The results of our study demonstrate that the main indicators characterizing arterial stiffness (pulse wave velocity, augmentation index, and ambulatory stiffness index) differed significantly in the compared groups. A statistically significant increase in the minimum, mean, and maximum PWV in the aorta was detected in children with familial hypercholesterolemia compared with these indicators in the control group. The study found a correlation between measures of arterial stiffness and levels of plasma serotonin, platelet serotonin, and membrane serotonin transporter levels.

Koba et al., in an experimental study, determined that LDL-cholesterol induces platelet aggregation by stimulating the release of 5-HT. LDL-cholesterol and serotonin act synergistically to enhance VSMC proliferation [51]. In our work, we identified a connection between the level of LDL-cholesterol in the blood plasma and the main indicators of serotonin.

We assume that the effect of serotonin on the vascular wall is secondary. Hypercholesterolemia promotes platelet hyperreactivity by direct interaction of oxidized low-density lipoprotein (oxLDL) with the platelet membrane by CD-36 receptors and signaling pathways including Src family kinases (SFKs), mitogen-activated protein kinases (MAPKs), and nicotinamide adenine dinucleotide phosphate (NADPH) oxidase [52]. A number of intracellular signaling mechanisms lead to the exocytosis of dense granules when platelets are activated. They release a wide range of molecules, such as ADP, ATP, Ca^2+^, and 5-HT. The secretion of dense granules leads to a feedback loop that enhances platelet aggregation and activation at the site of vascular injury because platelets themselves express appropriate receptors for these released substances, including 5-HT2A and 5-HT3 receptors [23].

The literature mainly describes the effects of acute post-infarction serotonin in adult patients, which differ from those in stable patients without atherosclerotic vascular disease. Serotonin affects platelet activation and aggregation, enhancing various pathways of primary (vascular-platelet) hemostasis [23,53]. Ziu et al. proved that in mice injected with 5-HT, the bleeding time from the tail was reduced [2]. Scientific evidence suggests that elevated plasma 5-HT levels are an independent risk factor for platelet hyperreactivity and, consequently, thrombosis [2]. We were not able to assess the effect of serotonin on the hemostatic system over time since our study was single-stage and time-limited.

5-HT is metabolized in the liver and eventually excreted from the body as 5-hydroxyindoleacetic acid [19]. The present study showed that plasma 5-HIAA levels were higher in patients with familial hypercholesterolemia than in controls, which correlated with plasma total cholesterol concentrations. There are no studies in the literature examining 5-HIAA in children with familial hypercholesterolemia.

The significant role of SERT and serotonin in the pathogenesis of atherosclerotic vascular lesions is currently confirmed by results obtained in the adult population [50]. For example, it has been proven that an increase in serotonin concentration is significantly associated with the progression of coronary artery disease and that this association is especially pronounced in young age groups [27]. Sarpogrelate, a 5-HT2A receptor antagonist, has also been found to improve outcomes in patients with peripheral artery disease [28]. Another study found a strong positive correlation between cIMT and 5-HT levels in adult patients with carotid atherosclerosis [54]. These data are consistent with our results obtained in the first study reported in the literature to determine plasma and platelet concentrations of SERT, serotonin, and 5-HIAA in children with familial hypercholesterolemia.

## 4. Materials and Methods

### 4.1. Recruitment of Study Groups 

**Figure 2:**
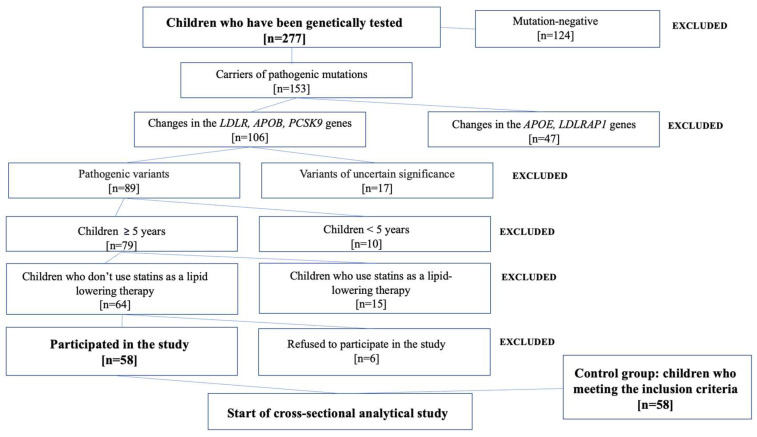
Flowchart of the study enrollment.

A cross-sectional study was performed with the participation of two pediatric groups in the Republican Center for Pediatric Lipidology. Children with familial hypercholesterolemia, included in the main group, were identified through cascade screening. A total of 153 people (parents) who were under the supervision of the lipidology center for adults were selected for the study. All adult patients were diagnosed according to the DLCN (Dutch Lipid Clinics Network Criteria). A total of 277 children of index patients underwent a genetic diagnosis using Next Generation Sequencing (NGS) on the IllumniaMiseqc platform using a custom Roche gene panel (Basel, Switzerland) containing coding sequences of the *LDLR*, *APOB*, *PCSK9*, *LDLRAP1*, and *APOE* genes. Pathogenic variants characteristic of familial hypercholesterolemia were identified in 106 children. A total of 58 children met the inclusion criteria (main group). Criteria for inclusion in the main group were: (1) the age from 5 to 17 years old, inclusive; (2) a genetically confirmed diagnosis of a heterozygous form of familial hypercholesterolemia; and (3) the presence of pathogenic variants in the *LDLR*, *APOB*, and *PCSK9* genes. Exclusion criteria: (1) an established diagnosis of a homozygous form of familial hypercholesterolemia; (2) the use of lipid-lowering drugs; (3) the appointment of drugs that alter or interact with the monoaminergic system; and (4) contraindications to ABPM. The control group included conditionally healthy children aged 5 to 17 years without CVD and with a TC level < 170 mg/dL [55]. A flowchart of the study enrollment is presented in Figure 2. All study participants and their legal representatives were provided with information sheets detailing the purpose, procedures, benefits, and potential risks of the study. Written informed consent was signed by all study participants or their legal representatives, and all of them provided their written informed consent prior to participation. The study was approved by the Research Ethics Committee of the Kazan State Medical University, Kazan, Russian Federation (Minutes No. 2 of 9 December 2019). This work was performed in accordance with the World Medical Association Code of Ethics (Declaration of Helsinki) for human experiments.

The study involved 116 children aged 5 to 17 years old. The main group included 58 patients with the diagnosis of a heterozygous form of familial hypercholesterolemia, and the control group included 58 children without CVD. Genetic testing was performed in 100% (n = 58) of children in the main group; pathogenic genetic variants in the *LDLR* gene were identified in 83% (n = 48) of patients, and *APOB* in 17% (n = 10). Pathogenic variants in the PCSK9 gene were not identified. The most common pathogenic variants in the *LDLR* gene were: c.986G>A (33% children—16/48), c.906C>G (27% children—13/48), c.1187-10G>A (12% children—6/48), and c.1202T>A (6% children—3/48). The remaining pathogenic variants detected in the LDLR gene were single and occurred in one or two patients with familial hypercholesterolemia. All children with pathogenic variants in the *APOB* gene (10/10) were heterozygotes with the c.10580G>A variant. The groups did not have statistically significant differences in age, gender, weight, or height. The number of boys and girls within the two groups was equal; the proportion of boys was 50% (29/58). The similarity of the groups according to the presented characteristics was achieved by the peculiarities of patient selection, which was similar to the methodology for selecting participants in the case-control study. Each patient from the main group was matched with a child from the control group who was similar in basic characteristics.

The main characteristics of the study groups are presented in Table 6. The body mass index (BMI) of children was assessed, taking into account their gender and age, using the Z-score criteria according to WHO recommendations [56]. More than half of the children had normal BMI values—79.3% (46/58) in the main group, but 15.5% (9/58) of them had mild malnutrition, and 5.2% (4/58) were overweight. Similar data were obtained in the control group. BMI in the range of normal values was registered in 75.8% (44/58) of children, 15.5% (9/58) of children were classified as undernourished, and 8.7% (5/58) were overweighted in controls.

The studied patient groups underwent a complete blood count, lipid profile analysis, biochemical blood test, and coagulogram.

Indicators of the lipid profile and biochemical blood analysis were determined in blood serum using a set of reagents from F. Hoffman La Roche (Basel, Switzerland) on an automatic analyzer Cobas 6000 (Roche, Basel, Switzerland).

### 4.2. Instrumental Examination

All children underwent ambulatory blood pressure monitoring (ABPM) with an assessment of arterial vascular stiffness. Indicators such as PWV, Aix, and AASI were measured during the instrumental study. Analysis of the values of the minimum PWV (PWVmin), mean PWV (PWV), and maximum PWV (PWVmax) obtained during ABPM revealed a statistically significant increase between the main and control groups (*p* < 0.001).

Ambulatory blood pressure monitoring with PWV assessment using the oscillometric method was performed using the BPLabVasotens system (Petr Telegin, Nizhny Novgorod, Russia). The following relation was used in the BPLab program to calculate the PWV: PWV = K × (2 × L)/RRWT, where K is the scale factor for normalizing the obtained PWV value; L is the length of the aortic trunk (in BPLab software (Version 06.02.01.15410), the length of the aorta is the distance from the upper edge of the sternum to the pubic bone); RRWT is the propagation time of the reflected wave.

cIMT was measured in both of the studied groups. The ultrasonic scanner HD11XE (Philips, Bothell, WA, USA) using a linear (3–12 MHz) probe was used. The technique was as follows: the child lied on the back and the head was slightly turned in the direction opposite to the studied artery, then the distal segment of the common carotid artery was located (about 1.5–2.0 cm proximal to its bifurcation) in longitudinal scanning in the B-mode. cIMT was measured along the posterior wall of the artery. One cursor was located on the adventitium–media border, the other on the intima–vessel lumen border. The assessment of cIMT was performed on both sides.

### 4.3. Determination of Serotonin and Serotonin Membrane Transporter

Blood sampling for patients in both groups was made in the morning after an overnight fast. Venous blood was collected in test tubes with ethylenediamineacetic acid and sodium citrate in a volume of 4 mL for laboratory studies. The obtained blood samples were stored at a temperature of −20 °C and delivered to the laboratory of the Children’s Republican Clinical Hospital for subsequent centrifugation and sample preparation within 1 h. 

The concentrations of serotonin and 5-HIAA in blood plasma and serotonin in platelets were determined by high-performance liquid chromatography (HPLC) with electrochemical detection, and the concentration of the membrane carrier of serotonin in platelets was determined by ELISA.

Venous blood obtained from study participants was centrifuged for 20 min at a temperature of +4 °C at a speed of 1000 rpm to determine the concentration of serotonin and 5-HIAA in blood plasma and serotonin in platelets. Then the plasma and formed elements were separated, and the platelet-rich supernatant was again centrifuged for 20 min at a temperature of +4 °C at a speed of 2500 rpm. The plasma and precipitate were transferred into two eppendorfs and stored at a temperature of −80 °C until analysis was performed. The content of 5-HT and 5-HIAA was determined by HPLC with electrochemical detection on an LC-304 T chromatograph (BAS, West Lafayette, IN, USA) and Gilson-307 on a Phenomenex C-18.4 μm, 150 mm × 4.6 mm column and an LC-4B amperometric detector with a TL cell—5 (BAS, West Lafayette, IN, USA). The measurements were made on a glassy carbon electrode at +0.85 V against an Ag/AgCl reference electrode. The volume of the injected sample was 20 μL. The mobile phase contained (g/L): potassium dihydrogen phosphate—9.56; sodium citrate—5.76; sodium 1-octanesulfonate—0.4 g; EDTA Na^+^—0.1; acetonitrile—8%. The mobile phase rate was 1.0 mL/min, and the pH was 3.0.

Two milliliters of venous blood were centrifuged at room temperature for 5 min at 200× *g* speed to determine the membrane transporter of serotonin. A total of 200 µL of platelet-rich plasma was transferred to another tube, and 800 µL of saline was added. Then it was centrifuged at a speed of 4500× *g* for 10 min at a temperature of +4 °C. The supernatant was removed, and 200 µL of distilled water was added to the platelet pellet. It was thoroughly mixed in a vortex and stored at −40 °C. The quantitative sandwich format Elisa, manufactured by Cloud-CloneCorp Lot: 221016400, was used to determine the membrane transporter of serotonin. Monoclonal antibodies were fixed on a microplate. Standards and samples were pipetted into wells according to the manufacturer’s instructions and bound to immobilized antibodies. Biotin-conjugated particles were added to the reaction wells after the removal of unbound substances. The avidin-enzyme reagent and substrate solution were added to visualize the reaction after a subsequent wash. The analysis was carried out on the BioTek immunoassay robotic system, and the calculation of the results was performed by the built-in software (Version 1.04).

### 4.4. Statistical Analysis

Analyses were conducted in the STATISTICA 6.0 program. The normal distribution of the trait was established at *p* > 0.05 (Shapiro–Wilk test). Nonparametric methods of statistical analysis were used otherwise. The arithmetic mean (M) and 95% confidence interval (CI) were calculated in the case of a normal distribution of the characteristic; the median (Me) was determined from measures of central tendency in the case of a distribution of the characteristic different from normal; from dispersion measures, the interquartile range (Q1–Q3, 25th and 75th percentile values). Relative frequencies of signs were presented as percentages (%), and the absolute values obtained and the total number of patients in the group (n/N) were indicated next to them. The significance of differences between groups was calculated using the Student’s *t*-test, chi-square test, Fisher’s exact test (in groups with a small number of participants), and Mann–Whitney U-test as well. The multiple comparison problem was solved using the Bonferroni correction. The relationship between two characteristics was analyzed using Spearman’s rank correlation.

## 5. Conclusions

The data we present indicate the relationship between high concentrations of serotonin, SERT, and markers of arterial stiffness. As a result of our study, we identified different levels of the membrane transporter of serotonin, 5-HT, and 5-HIAA in children with familial hypercholesterolemia in comparison with the control group of relatively healthy children. The results obtained suggest the involvement of serotonin and SERT in the process of vascular remodeling in children with familial hypercholesterolemia. Considering that these indicators differ significantly in patients with realized CVD risks, such as coronary heart disease, we suppose that serotonin, 5-HIAA, and membrane transporters can be used as laboratory markers of atherosclerotic changes in the vascular wall and also become new therapeutic targets for the treatment and prevention of the progression of atherosclerotic vascular lesions in children and adults. A follow-up study is planned to confirm our findings.

## Figures and Tables

**Table 1:** Results of lipid profiles of children with and without familial hypercholesterolemia.

	Main Group		Control Group		*p* ^1^
	Mean	CI	Mean	CI	
TC, mg/dL ^2^	259.02	150.77–371.14	150.77	96.65–204.9	<0.001
HDL-cholesterol, mg/dL	57.99	34.79–77.32	61.86	42.53–85.05	0.004
LDL-cholesterol, mg/dL	185.57	81.19–289.95	85.05	46.39–119.85	<0.001
TG, mg/dL	87.5	43.75–131.25	70	17.5–131.25	0.003

^1^ *p*—the level of statistical significance of differences. ^2^ TC—total cholesterol; HDL-cholesterol—high density lipoproteins; LDL-cholesterol—low density lipoproteins; TG—triglycerides.

**Table 2:** Results of ambulatory blood pressure monitoring in patients with and without familial hypercholesterolemia.

Index	Main Group		Control Group		*p* ^1^
	Median	Q_1_–Q_3_	Median	Q_1_–Q_3_	
PWV min, m/s ^2^	4.1	3.5–5.6	3.3	2.8–4.1	<0.001
PWV, m/s	6.9	5.5–8	4.4	3.7–5.1	<0.001
PWV max, m/s	8.5	7.3–10.6	6.6	5.8–7.4	<0.001
AIxmin, %	−73.5	−82–−67	−75	−84–−69	0.179
Aixmean, %	−39.5	−47–−35	−51	−58–−45	<0.001
AIxmax, %	9.5	3–30	7.5	−3–26	0.179
AASI	0.5	0.2–0.8	0.4	0.2–0.7	<0.001

^1^ *p*—the level of statistical significance of differences. ^2^ PWV—pulse wave propagation velocity; AIx—augmentation index per day; AASI—ambulatory stiffness index.

**Table 3:** Values of serotonin, its metabolites, and transporter in children with and without familial hypercholesterolemia (controls).

	Main Group		Control Group		*p* ^1^	q
	Median	Q_1_–Q_3_	Median	Q_1_–Q_3_		
Plasma 5-HT (pmol/mL) ^2^	96.7	24.2–175.2	66.2	42.4–79.2	<0.001	<0.001
Platelets 5-HT (pmol/10^9^ platelets)	0.03	0.01–0.5	0.02	0.01–0.03	0.007	0.014
	**Mean**	**CI**	**Mean**	**CI**		
Platelets 5-HT (pmol/mL)	8,530,370	2,326,366–14,734,712	6,235,403	723,560–11,747,293	<0.001	0.004
SERT, ng/mL	0.04	0.02–0.07	0.03	0.01–0.06	<0.001	0.002
5-HIAA in blood plasma (pmol/mL)	27.2	14.2–40.1	20.9	8.4–33.5	<0.001	<0.001

^1^ *p*—level of statistical significance of differences; q—Bonferroni correction. ^2^ 5-HT—serotonin; 5-HIAA—5-hydroxyindoleacetic acid; SERT—membrane serotonin transporter.

**Table 4:** Correlation between basic serotonin parameters and instrumental markers of arterial stiffness in children with and without familial hypercholesterolemia.

	cIMT ^1^		PWVmin		PWV		PWVmax		AASI	
	*ρ* ^2^	*p*	*ρ*	*p*	* ρ *	*p*	* ρ *	*p*	* ρ *	*p*
5-HT in plasma ^3^	0.5	<0.001	0.4	<0.001	0.6	<0.001	0.5	<0.001	0.3	<0.001
Platelets 5-HT	0.4	<0.001	0.3	0.001	0.5	<0.001	0.4	<0.001	0.3	<0.001
5-HT in 1 platelet	0.3	<0.001	0.3	0.002	0.4	<0.001	0.4	<0.001	0.4	<0.001
5-HIAA	0.4	<0.001	0.2	0.0026	0.4	<0.001	0.4	<0.001	0.3	0.001
SERT	0.4	<0.001	0.3	<0.001	0.5	<0.001	0.4	<0.001	0.3	<0.001
5-HT in plasma ^3^	0.5	<0.001	0.4	<0.001	0.6	<0.001	0.5	<0.001	0.3	<0.001

^1^ cIMT—carotid intima-media thickness; PWV—pulse wave propagation velocity; AASI—ambulatory stiffness index. ^2^ *ρ*—Spearman’s correlation coefficient; *p*—level of statistical significance of differences. ^3^ 5-HT—serotonin; 5-HIAA—5-hydroxyindoleacetic acid; SERT—membrane serotonin transporter.

**Table 5:** Correlation between basic serotonin parameters and lipid profile results in children with and without familial hypercholesterolemia.

	TC ^1^		LDL-Cholesterol		HDL-Cholesterol	
	*ρ* ^2^	*p*	* ρ *	*p*	* ρ *	*p*
5-HT in plasma ^3^	0.5	<0.001	0.5	<0.001	−0.2	0.03
Platelets 5-HT	0.3	<0.001	0.4	<0.001		
5-HT in 1 platelet	0.3	0.003	0.3	<0.001		
5-HIAA	0.5	<0.001	0.4	<0.001	−0.3	<0.001
SERT	0.3	0.003	0.3	0.005		

^1^ TC—total cholesterol; LDL-cholesterol—low-density lipoproteins; HDL-cholesterol—high-density lipoproteins. ^2^ *ρ*—Spearman’s correlation coefficient; *p*—level of statistical significance of differences. ^3^ 5-HT—serotonin; 5-HIAA—5-hydroxyindoleacetic acid; SERT—membrane serotonin transporter.

**Table 6:** The main characteristics of the study groups.

	Main Group		Control Group		*p* ^1^
	Mean	CI	Mean	CI	
Age, years	10.5	2.8–18.1	10.5	2.8–18.1	1
Height, cm	143.1	98.4–177.7	141.7	95.4–187.9	0.758
Weight, kg	37.7	6.1–69.3	38.2	1.8–74.6	0.868
z-score BMI	−0.18	−1.9–1.5	−0.07	−2.1–2	0.544

^1^ *p*—the level of statistical significance of differences.

## Data Availability

All data are contained within this article.
